# Protocol for generating protoplasts from the leafcutter ant symbiotic fungus *Leucoagaricus gongylophorus*

**DOI:** 10.1016/j.xpro.2025.104331

**Published:** 2026-01-09

**Authors:** Ayoub Stelate, Jonathan Z. Shik

**Affiliations:** 1 Section for Ecology and Evolution, Department of Biology, University of Copenhagen, København, Denmark; 2 Smithsonian Tropical Research Institute, Apartado Postal Balboa, Ancon 0843-03092, Panama

**Keywords:** Cell Biology, Cell isolation, Microscopy, Environmental sciences, Evolutionary biology

## Abstract

We present a protocol to isolate viable protoplasted cells of the basidiomycete fungal mutualist (*Leucoagaricus gongylophorus*) obligately farmed by leafcutter ants. We describe steps for culturing actively growing mycelia, enzymatic digestion of fungal cell walls under osmotic stabilization, and purification of protoplasts (plasma membrane enclosing cytoplasm and organelles). We then detail procedures for assessing viability and wall removal using microscopy and fluorescent staining.

## Highlights

• Steps for enzymatic digestion of *Leucoagaricus gongylophorus* cell walls
• Instructions for purification and stabilization of isolated fungal protoplasts
• Guidance on microscopy validation and quantitative protoplast assessment

## Abstract (editor highlights)

Publisher’s note: Undertaking any experimental protocol requires adherence to local institutional guidelines for laboratory safety and ethics.

## Graphical abstract

**Figure:**
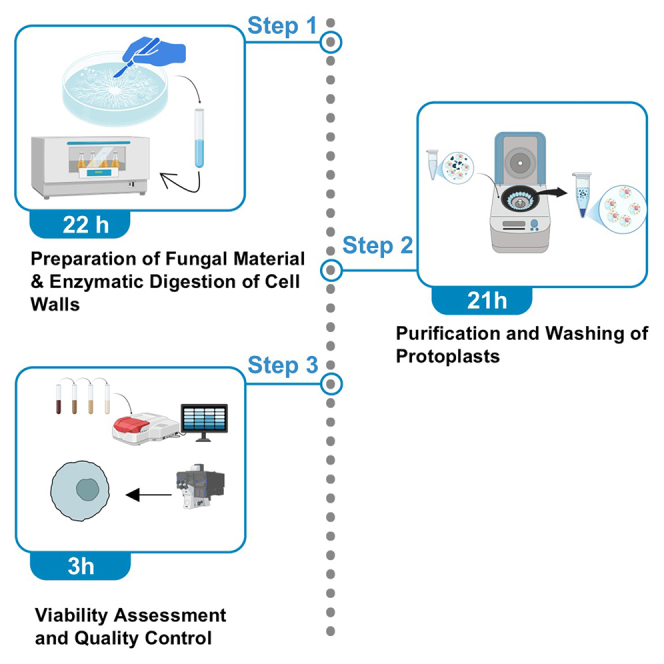

## Before you begin

Axenic *in vitro* cultures are first prepared using clusters of swollen cells (gongylidia bundled into staphylae) freshly isolated from fungal gardens of laboratory-maintained leafcutter ant colonies. After isolation, the inoculum is grown on agar plates containing Potato Dextrose Agar (PDA) in the dark at 25°C for three weeks (or until the growth area is approximately 0.5 cm in diameter). Under such conditions, the fungus culture typically develops actively growing hyphal fronts that can be excised for protoplast isolation. Protoplasting methods perform best with tissue from incipient and vigorously growing cultures. The excised hyphae and gongylidia are then entered into the cell wall digestion protocol.

### Innovation

Protoplast isolation is an established approach for studying fungal cell biology and enabling genetic manipulation.^1^^,^^2^ Yet, most protocols have been optimized for model ascomycetes^3^^,^^4^ or a limited set of basidiomycetes.^5^ To date, no reproducible strategy has been available for generating protoplasts from *Leucoagaricus gongylophorus*, the fungal cultivar obligately farmed by leafcutter ants. Thus, while leafcutter ants are model systems for evolutionary and ecological studies,^6^^,^^7^ as well as important agricultural pests,^8^ the mechanisms underlying the cultivar’s coevolved crop productivity have remained poorly understood.

First, we define an enzymatic protocol tailored to the chitin- and glucan-rich cell wall composition of *L. gongylophorus*, balancing cell wall degradation with osmotic stabilization to minimize lysis of the protoplasts. Second, we develop streamlined purification steps and quality control assays, including viability staining and cell wall integrity testing to ensure reproducibility across preparations.

This protocol therefore expands the experimental toolkit for *L. gongylophorus*, providing direct access to viable fungal cells without cell walls. By removing the rigid cell wall, this approach overcomes a major barrier to downstream work since the wall normally blocks uptake of DNA, dyes, and large molecules and limits single-cell manipulation. Beyond its immediate use for genetic transformation and single-cell analysis, the approach demonstrates how protoplast isolation can be adapted to other challenging non-model basidiomycetes. By enabling cellular and molecular studies of the symbiotic crop fungus of leafcutter ants, this protocol enables cutting edge tools to explore mechanisms of symbiotic nutrient exchange and establishes a range of biotechnological applications.

### Institutional permissions

This protocol uses the fungal symbiont *Leucoagaricus gongylophorus* isolated from Atta ant gardens. All fungal isolations and subsequent laboratory manipulations were conducted under biosafety level 1 (BSL-1) conditions at the University of Copenhagen. Work involving imported ant-fungus material was performed under issued permits for the handling of non-pathogenic agricultural microbial symbionts. Users should consult local regulations and obtain any required institutional and import permissions before collecting, transporting, or culturing fungal symbionts.

### Preparation of fungal cultures

**Timing: 3 weeks**

1. Culture *L. gongylophorus* on potato dextrose agar (PDA) medium under sterile, axenic conditions until the colony margin shows active hyphal growth (approximately for three weeks).

***Note:*** Young, actively growing hyphae (i.e. a dense, white, and fluffy appearance at the colony center, in contrast to the flatter, more translucent at the margin) can be more readily digested than old or melanized tissue.

**CRITICAL:** Avoid overgrown or nutrient-depleted plates as such material yields fewer protoplasts with lower viability.

2. Just prior to protoplast isolation, wash the harvested tissue gently with osmotic stabilization buffer to remove excess media and extracellular matrix material.

**Pause point:** Washed mycelia can be kept in osmotic buffer at 4°C for up to 2 h before proceeding to enzymatic digestion.

### Preparation of buffers and enzyme mixture

**Timing: 1 h**

3. Prepare fresh osmotic stabilization buffer and pre-equilibrate to the incubation temperature (25°C) required for digestion.

***Note:*** Osmotic buffer can be prepared in larger batches (e.g., 50 mL) and stored at 4°C for up to 1 week before use

**CRITICAL:** Verify osmotic concentration carefully by observing a small test aliquot for stability (e.g., lack of immediate swelling or lysis) before committing large samples or downstream assays; insufficient osmotic stabilization can cause premature protoplast lysis.

4. Prepare 2 mL of enzyme solution freshly for each digestion, which is sufficient for 2 mg of collected mycelial tissue (based on our experiments).

***Note:*** Prepare the digestion cocktail immediately before use.

5. Store the mixture at 25°C until it is added to fungal material.

**CRITICAL:** Enzyme activity decreases with repeated freeze–thaw cycles. It is best to prepare only the amount needed for immediate use and avoid freezing leftover mixtures for later.

### Preparation of equipment

**Timing: 4 h**

6. Sterilize and assemble required consumables, including filtration meshes or sterile nylon cloth, wide-bore pipette tips, and centrifuge tubes.

**Pause point:** Equipment can be prepared in advance and stored at 20°C until use.

7. Calibrate centrifuges and set up a shaker or incubator to the appropriate digestion temperature and agitation speed.

**CRITICAL:** Confirm rotor compatibility with the sample volume and tube type to avoid sample loss during centrifugation.

## Key resources table

**Table:** 

REAGENT or RESOURCE	SOURCE	IDENTIFIER
**Biological samples**		

*Leucoagaricus gongylophorus*	*Atta colombica* ant colony (Panama)	EG20211101-2

**Chemicals, peptides, and recombinant proteins**		

Chitinase from *Streptomyces griseus*	Sigma-Aldrich	Cat# 9001-06-3
Cellulase from *Trichoderma sp.*	Sigma-Aldrich	Cat#9012-54-8
Driselase from *Basidiomycetes sp*.	Sigma-Aldrich	Cat#85186-71-6
β-Glucuronidase Type HP-2	Sigma-Aldrich	Cat#9001-45-0
FM4-64 Dye	Invitrogen	Cat# T3166
Calcofluor White stain (CFW)	Sigma-Aldrich	Cat#18909
Potato Dextrose Agar (PDA)	Chemsolute	Cat#8992.0500
Bovin Serum Albumin (BSA)	Sigma-Aldrich	Cat# 9048-46-8
MES hydrate	Sigma-Aldrich	Cat#1266615-59-1
Dimethyl sulfoxide (DMSO)	Sigma-Aldrich	Cat#D2650
D-Sorbitol	Sigma-Aldrich	Cat#50-70-4
Phosphate Buffered Saline (PBS)	Amresco	Cat#E404-200TABS
Trypan blue	Thermo Fisher Scientific	Cat#15250061
D-Mannitol	Sigma-Aldrich	Cat#69-65-8
Syto18 Dye	Invitrogen	Cat# S32703

**Other**		

pH meter	Frederiksen Scientific	542600
Millex PVDF syringe filter (0.22 μm)	Sigma-Aldrich	SLGVR33RB
Nylon mesh filter (40 μm)	Sigma-Aldrich	CLS431750
Centrifuge	Eppendorf	5430
Ocelloscope	BioSense Solutions	USC.73.0109R002N0010
Inverted fluorescence microscope	Leica	Stellaris 8

## Materials and equipment

**Table:** Osmotic Buffer A (1.2 M Mannitol, pH 6)

Reagent	Final concentration	Amount for 20 mL
D-Mannitol	1.2 M	4.37 g
BSA	0.2% (W/V)	40 mg
MES hydrate, pH 6	0.1 M	390.84 mg
Sterile distilled water	—	up to 20 mL

Storage: Store at 4°C for up to 1 week. Warm to 20°C before use.

**Table:** Osmotic Buffer B (1.2 M Sorbitol, pH 6)

Reagent	Final concentration	Amount for 20 mL
Sorbitol	1.2 M	4.37 g
BSA, pH 6	0.2%(W/V)	40 mg
MES hydrate, pH 6	0.1 M	390.84 mg
Sterile distilled water	—	up to 20 mL

Storage: Store at 4°C for up to 1 week. Warm to 20°C before use.

***Note:*** Both mannitol and sorbitol-based osmotic buffers performed equivalently in our hands.

• Enzyme Solution for Cell Wall Digestion

Prepare fresh enzyme solution just before using. Dissolve enzymes in Osmotic Buffer A or B immediately prior to use. Adjust the pH to 6. Filter-sterilize with 0.22 μm filter before adding to fungal suspension.

**Table:** Enzyme Cocktail for Protoplast Isolation

Enzyme	Concentration in working solution	Volume for 2 mL cocktail
Chitinase (*S. griseus*)	1.25 mg/mL	2.5 mg
Cellulase (*Trichoderma sp.*)	18.4 mg/mL	36.8 mg
Driselase	20 mg/mL	20 mg
β-Glucuronidase Type HP	250 μL/mL	500 μL
Osmotic buffer (A or B)	—	up to 2 mL

Storage: Prepare fresh before each usage; do not store enzyme cocktail.

***Note:*** This volume is adjusted for 2 mg of wet mass mycelia.

### Staining solutions

#### Calcofluor White (CFW)

**Figure 1:**
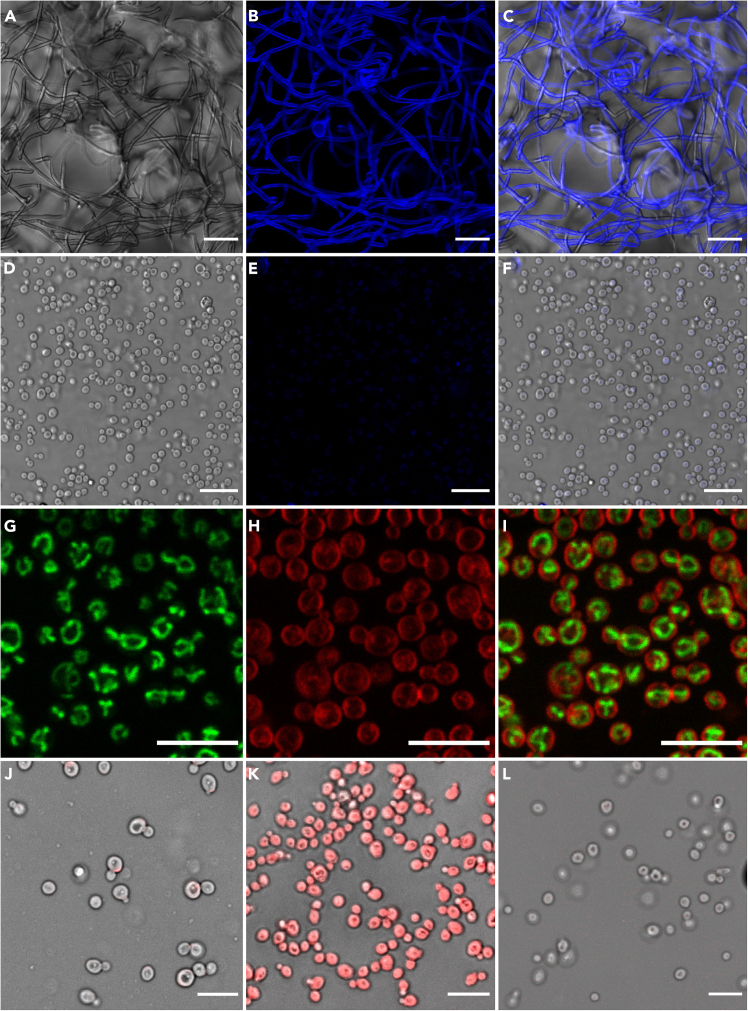
Representative microscopy images showing the successful isolation, cell wall removal, and viability assessment of *L. gongylophorus* protoplasts (A) Brightfield channel image of *L. gongylophorus* fungus prior to enzymatic digestion. (B) Calcofluor White (CFW) channel showing chitin-rich cell walls of the same hyphae. (C) Overlay of brightfield and CFW channels showing intact cell walls before digestion. (D) Brightfield image of isolated *L. gongylophorus* protoplasts following enzymatic treatment. (E) CFW staining of the isolated protoplasts showing absence of cell wall fluorescence. (F) Overlay of brightfield and CFW channels confirming successful cell wall removal. (G) Syto18 staining revealing mitochondrial distribution within viable protoplasts. (H) FM4-64 staining visualizing the plasma membrane of the same protoplasts. (I) Overlay of Syto18 and FM4-64 channels highlighting distinct mitochondrial and membrane localization. (J) Viability assessment of isolated protoplasts using Trypan Blue staining (viable protoplasts do not take up the dye). (K) Positive control of fixed protoplasts (4% paraformaldehyde) showing uniform Trypan Blue uptake. (L) Unstained protoplasts imaged in osmotic buffer were used as a negative control to confirm that the dye binds specifically. Scale bars: (A–C) 40 μm; (D–F) 20 μm; (G–L) 10 μm.

***Note:*** This diluted solution can be stored in the dark at 4°C for up to 6 months.

• Prepare a 1:100 dilution of Calcofluor White stock in osmotic buffer to achieve a final working concentration of 10 μg/mL.
• Incubate protoplasts for 1–2 min at 25°C, wash once with PBS.
• Observe under UV excitation (Ex: 365–405 nm, Em: 435–485 nm) (Figures 1B and 1E).

#### FM4-64 (plasma membrane stain)

Dissolve FM4-64 in DMSO to prepare a 1 mM stock solution and store at −20°C, protected from light, for up to 1 year. For staining:

• dilute the stock to a final concentration of 5 μM in osmotic buffer.
• Incubate protoplasts at 25°C for 3 min, wash once with osmotic buffer.
• Visualize under fluorescence microscopy (Ex: 558 nm, Em: 734 nm) (Figure 1H).

***Note:*** The working solution should be prepared fresh each time and not stored.

#### SYTO 18 (mitochondrial/nucleic acid stain)

SYTO 18 is supplied as a 5 mM stock in DMSO and should be stored at −20°C, protected from light, for up to 1 year. To stain protoplasts:

• Dilute the stock to a final concentration of 10 μM in osmotic buffer.
• Incubate cells at 25°C for 3–5 min, and image with fluorescence microscopy (Ex: 480–500 nm, Em: 530–550 nm) (Figure 1G).

***Note:*** Working solutions should be prepared immediately before use.

#### Trypan blue (viability stain)

• Prepare a 0.4% (w/v) Trypan Blue solution by dissolving 0.4 g of Trypan Blue powder in 100 mL osmotic buffer.
• Sterilize the solution using a 0.22 μm filter.
• For viability assessment, mix equal volumes of protoplast suspension and Trypan Blue solution, then observe stained (non-viable) and unstained (viable) protoplasts under a light microscope.

***Note:*** When visualized using a fluorescence microscope, non-viable protoplasts exhibit red fluorescence due to intracellular accumulation of the dye (Figure 1H) (excitation at ∼580 nm and emission at ∼680 ±50 nm), while viable protoplasts remain non-fluorescent (Figure 1J). The solution can be stored in the dark at 4°C for up to 6 months.

### Filtration setup

• Use sterile nylon mesh filters (40 μm) to separate protoplasts from undigested mycelia.
• Pre-wet filters with osmotic buffer before use to minimize sample loss.

## Step-by-step method details

### Preparation of fungal material

**Timing: 15 min**

This section describes the collection and preparation of mycelial tissue from *Leucoagaricus gongylophorus* for enzymatic digestion.

1. Excise growing hyphae from three weeks old *Leucoagaricus gongylophorus* cultures (approximately 15 mg harvested) using sterile tools.
2. Transfer hyphal culture into sterile osmotic buffer to remove residual medium.
3. Wash tissue fragments gently until no visible medium remains, typically yielding 2 mg wet mass of cleaned fungal material.

***Note:*** Use aseptic technique in all steps to avoid contamination.

Troubleshooting: See troubleshooting—“Contamination or poor starting material.”

### Enzymatic digestion of cell walls

**Timing: 21 h**

This section describes preparation of the enzyme cocktail and digestion of cell walls to generate spherical, wall-free protoplasts.

4. Prepare a fresh enzyme cocktail by combining enzymes in osmotic buffer immediately before use. 2 mL of enzyme mix, which is optimized for 2 mg wet mass mycelial tissue.

***Note:*** Confirm that the pH is 6.0; adjust if necessary.

5. Transfer washed hyphal fragments into a digestion vessel and add the enzyme cocktail until tissue is fully submerged.
6. Incubate under 25°C while shaking at 200 rpm.
7. Monitor digestion microscopically at regular intervals (e.g., every 4–6 h) to track the progression of cell wall digestion.

**Figure 2:**
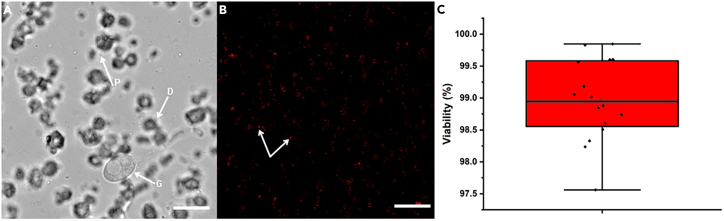
Monitoring enzymatic digestion and viability of *Leucoagaricus gongylophorus* protoplasts (A) Time-course light microscopy image showing *L. gongylophorus* hyphae during enzymatic digestion at a 6-hour interval. Arrows indicate emerging protoplasts (P), hyphal debris generated during wall degradation (D), and partially digested gongylidia (G). (B) Trypan Blue exclusion assay of isolated protoplasts. Arrows indicate non-viable protoplasts that have taken up the dye, whereas viable protoplasts exclude the stain and retain a clear cytoplasm with dye restricted to the membrane surface. (C) Box plot summarizing protoplast viability quantified across 15 independent microscopy fields (Average of 573 protoplasts per 250 μm^2^ field). The central line denotes the mean viability value, whiskers represent standard deviation, and individual data points reflect measurements from each field. The overall viability averaged ∼98% across all samples. Scale bar is 40 μm.

***Note:*** After 6 h, hyphae should show early wall softening, swelling, and partial digestion; an example image of this stage is provided (Figure 2A). Continue monitoring until a substantial proportion of spherical, wall-free protoplasts appear (typically 80%–90% of visible structures).

**CRITICAL:** Stop digestion once protoplast yield is sufficient to prevent loss of viability.

Troubleshooting: See troubleshooting—“Incomplete digestion or over-digestion.”

### Protoplast release and separation

**Timing: 20 min**

This step detaches protoplasts from partially digested hyphae and removes undigested debris.

8. If needed, release protoplasts from partially digested tissue by gentle mechanical dispersion (e.g., pipetting or agitation).
9. Pass the suspension through a sterile 40 μm nylon mesh filter to remove undigested mycelial debris.
10. Collect the filtrate containing protoplasts for purification.

***Note:*** Pre-wet filters with osmotic buffer to minimize sample loss.

Troubleshooting: See troubleshooting—“Loss of protoplasts during filtration.”

### Purification and washing of protoplasts

**Timing: 20 min**

This step removes enzymes and soluble digestion byproducts while maintaining protoplast integrity.

11. Clarify the filtrate by low-force centrifugation (e.g., 100 × g, 1 min) with swinging-bucket rotor, which prevents compression of protoplasts against hard tube surfaces. or gravity sedimentation to separate protoplasts from soluble debris.
12. Carefully remove supernatant and gently resuspend the pellet in fresh osmotic buffer. The pellet appear as a small, pale, translucent pellet forming at the bottom of the tube.
13. Repeat washing steps 2–3 times until residual enzymes are minimized.

Troubleshooting: See troubleshooting—“Protoplast lysis during wash.”

### Viability assessment and quality control

**Timing: 3 h**

This section describes microscopy-based validation of cell wall removal, plasma membrane integrity, mitochondrial activity, viability testing, and quantitative determination of protoplast concentration.

14. Determine total protoplast concentration using an Ocelloscope system.
  a. Prepare a series of tenfold serial dilutions of the protoplast suspension in the appropriate osmotic stabilizer (e.g., 1.2 M Sorbitol), each in triplicate.
  b. Transfer 200 μL from each dilution into a 96-well microplate and measure using the Ocelloscope’s cell-counting module.
  c. Identify the dilution that provides optimal detection and minimal overlap between cells; in this case, the 1:1000 dilution yielded the most reliable counts.
    ***Note:*** For example, in two independent samples, the Ocelloscope detected ∼5.8 × 10^4^–7.3 × 10^4^ cells per 200 μL at the 1:1000 dilution, corresponding to ∼5.9 × 10^8^–6.7 × 10^8^ total protoplasts per 2 mL of the original suspension. Ocelloscope-based counting is optional and can be substituted with a hemocytometer or automated cell cytometer.
    **CRITICAL:** High protoplast density or residual debris may interfere with automated detection. If needed, increase the dilution factor or gently filter the suspension through a 40 μm mesh (see troubleshooting: “Ocelloscope detection interference due to debris or high density”).

15. Visualize protoplast integrity using a 40× objective on a confocal laser scanning microscope with cell wall–specific stains (CWF).
  a. Dilute CFW 1:100 in osmotic buffer, incubate cells for 1–2 min at 25°C, wash once, and image under UV excitation.

***Note:*** Intact protoplasts should show little or no cell-wall staining compared with untreated hyphae (Figure 1E).

16. Stain protoplasts with Trypan Blue (0.4%) to estimate yield and viability.
  a. Mix an equal volume of protoplast suspension with Trypan Blue Figure 1J).

***Note:*** When imaged by confocal microscopy, Trypan Blue can be detected via red fluorescence (Ex ∼580 nm, Em ∼650–750 nm), enabling clear discrimination between non-viable (Figure 1K) and viable (Figure 1J) protoplasts within the same field (Figure 2B).

Troubleshooting: See troubleshooting—“Low viability despite intact morphology.”

17. Samples can also be assessed for plasma membrane condition and mitochondrial activity:
  a. Stain protoplasts with FM4-64 (final concentration 5 μM) for 3 min at 25°C. As described in step 14, wash sample once, then image (Ex: 558 nm, Em: 734 nm) (Figure 1H).
  b. Stain protoplasts with Syto18 (final concentration 10 μM from a 5 mM stock). Incubate 3–5 min at 25°C, then image mitochondrial fluorescence (Figure 1G).

## Expected outcomes

This protocol yields viable protoplasts from *Leucoagaricus gongylophorus* suitable for downstream applications such as transformation, regeneration assays, or live-cell imaging. From approximately 2 mg of fresh mycelial tissue digested in 2 mL of enzyme cocktail, researchers can expect to obtain between 5.88 × 10^8^ cells/2 mL and 6.74 × 10^8^ cells/2 mL total protoplasts numbers. Protoplasts appear as spherical, cell wall–free structures under light microscopy Figures 1D–1F), distinct from undigested hyphal fragments (Figures 1A–1C). Calcofluor White (CFW) staining confirms the successful removal of the cell wall and the integrity of the protoplast cell membrane. Calcofluor White staining should yield little or no fluorescence in viable protoplasts (Figure 1E), indicating effective digestion of chitin-rich cell walls. Trypan Blue exclusion allows rapid assessment of protoplast viability, with viable protoplasts excluding the dye and remaining clear (Figure 1J). Based on Trypan Blue quantification across two biological replicates, protoplast preparations generated using this protocol consistently show approximately 98% viability (Figures 2B and 2C). Membrane staining with FM4-64 produces strong plasma membrane labeling (Figure 1H), while SYTO 18 highlights mitochondrial or nucleic acid signals (Figure 1G), providing additional confirmation of cellular integrity. Protoplast suspensions prepared using this protocol remain stable for several hours in osmotic buffer and can be concentrated, washed, and directly used for other studies. High-quality images acquired by differential interference contrast (DIC) or fluorescence microscopy should show a homogeneous population of spherical protoplasts with intact membranes, low background debris, and consistent staining patterns. These outcomes demonstrate that this protocol provides a reproducible and robust approach for generating *L. gongylophorus* protoplasts for downstream experimental applications.

## Quantification and statistical analysis

Protoplast concentration was quantified using the Ocelloscope system. A 1:1000 dilution of the protoplast suspension was loaded in triplicate (200 μL per well), and cell counts were determined automatically. The average counts were 58,769 ± 11,274 cells per 200 μL for the first sample and 67,393 ± 3,216 cells per 200 μL for the second. These correspond to approximately 5.9 × 10^8^ and 6.7 × 10^8^ protoplasts per 2 mL of the original undiluted suspension, respectively. These measurements confirm that the protocol yields sufficient cell density for downstream applications such as genetic transformation and live-cell assays.

Viability percentage was quantified using 15 independent microscopy fields, each corresponding to a 250 μm^2^ area. Protoplasts (Figure 2B) were manually segmented and counted using the Cell Counter plugin in ImageJ, with viable cells identified by exclusion of Trypan Blue and non-viable cells identified by dye uptake. The viability percentage for each image was calculated as the proportion of unstained cells relative to the total protoplast count. Box plots (Figure 2C) were generated in OriginPro.

## Limitations

Success of this protocol depends on the condition of the *in vitro* fungal culture. Young and actively growing mycelia work best, while older or stressed cultures often yield few protoplasts because the cell walls become thicker and more resistant to digestion. Another limitation is the variability in enzyme activity between batches, if the cocktail is not fresh or the enzymes have lost activity during storage, digestion can be incomplete. Environmental factors such as temperature, shaking speed, and pH also mediate success as small deviations from the planned protocol can slow down digestion or cause the protoplasts to lyse. Handling is another critical point: too much pipetting or centrifuging at excessive speeds will damage the protoplasts. Even when everything works well, the protoplasts are not stable for long periods. They tend to lose viability within a few hours, which limits the time available for downstream experiments. Thus, while the protocol is reliable for producing viable protoplasts for microscopy and short-term assays, it may need additional adjustments if large-scale regeneration or transformation experiments are planned.

## Troubleshooting

### Problem 1

Contamination of cultures or poor starting material (Steps 1–3).

### Potential solution

This can occur if the staphylae are not handled under strictly sterile or axenic conditions, or if the material is left to grow for too long before excision. To minimize this issue, use staphyla freshly isolated from the fungus garden of leafcutter colonies and allow the culture to grow for three weeks. At this point, hyphae and gongylidia can be excised and used for digestion. If contamination persists, fresh staphyla can be reisolated from the fungal garden and the process repeated.

### Problem 2

Incomplete digestion of hyphae or low protoplast yield (Steps 4–7).

### Potential solution

Sometimes digestion is inefficient, giving very few protoplasts. This is usually linked to enzyme activity, culture age, or pH imbalance. Ensure the enzyme cocktail is freshly prepared and precisely adjusted to pH 6. Enzyme activity can vary between manufacterer lots, so if the yield is low, prepare fresh stocks or try another batch. With older, densely growing mycelia, extend incubation slightly, but check regularly to avoid over-digestion.

### Problem 3

Over-digestion and loss of protoplast viability (Steps 6 and 7).

### Potential solution

Over-digestion of tissues can yield fragile protoplasts with low viability. If this happens, stop digestion as soon as sufficient spherical protoplasts are visible. Lowering shaking speed helps reduce lysing. If the problem continues, shorten incubation duration or decrease enzyme concentrations to better match the quantity of fungal material.

### Problem 4

Loss of protoplasts during filtration (Steps 8 and 9).

### Potential solution

Protoplasts can be lost during filtration, especially if they adhere to the nylon mesh. To reduce this, pre-wet the mesh with osmotic buffer and filter slowly without applying pressure, since forcing the suspension through the mesh can damage cells. If recovery is still poor, switch to a 60 μm mesh even if this reduces sample purity and includes more tissue debris.

### Problem 5

Protoplast lysis during washing (Steps 11–13).

### Potential solution

Protoplasts can lyse during washing, mostly due to excessive centrifugation forces. Since excessive speeds can cause protoplast rupture, use low-speed centrifugation (i.e., 100 × g) or even gravity sedimentation. Additionally, gently resuspend protoplasts with wide-bore tips (1 mL) or cut pipette tips. If lysis persists, increase the osmotic stabilizer slightly, for example by increasing mannitol to 1.3 M.

### Problem 6

Low viability despite intact morphology (Steps 14–16).

### Potential solution

Sometimes protoplasts look intact but show low viability after staining. This usually indicates that the osmotic buffer is not fresh or that residual enzymes remain from previous isolation. Be sure to prepare fresh buffer and wash thoroughly to remove even trace amounts of enzymes. If viability remains low, shorten the digestion time or reduce enzyme concentrations, because extended exposure can weaken cells even if they appear functional under the microscope.

### Problem 7

Ocelloscope detection interference due to debris or high density (Step 14).

### Potential solution

When quantifying protoplasts with an Ocelloscope, I noticed that the presence of undigested debris can interfere with object recognition, leading to an overestimation of cell numbers. Similarly, a high density of protoplasts in the sample may cause overlapping signals and reduce counting accuracy. To minimize these issues, the suspension should be diluted so that individual protoplasts are well separated in the field of view. Allowing the suspension to settle or gently filtering it before measurement also helps reduce background noise. In my case, a 1:1000 dilution and 200 μL volume per well provided consistent results, but triplicate runs were still necessary to account for minor variations caused by uneven distribution or optical interference.

## Resource availability

### Lead contact

Further information and requests for resources and reagents should be directed to and will be fulfilled by the lead contact, Ayoub Stelate (ayoub.stelate@bio.ku.dk).

### Technical contact

For technical specifics on executing the protocol, Ayoub Stelate (ayoub.stelate@bio.ku.dk) will provide support to ensure its correct implementation.

### Materials availability

This study did not generate new unique reagents and materials.

### Data and code availability

• All data are available from the lead author upon request.
• This article does not report original code.
• Any additional information required to reanalyze the data reported in this article is available from the lead contact upon request.

## Acknowledgments

We would like to thank Henrik Hjarvard de Fine Licht and Andi Wilson for granting access to the Ocelloscope facility used for protoplast counting. Some cartoon materials were adopted from BioRender.com for the graphs. J.Z.S. was supported by a Semper Ardens: 10.13039/100001438Accelerate Grant (CF22-0664) from the 10.13039/100020038Carlsberg Foundation, Denmark (https://www.carlsbergfondet.dk).

## Author contributions

The study was designed by A.S. with inputs from J.Z.S. The experimental work was carried out by A.S. The first draft of the manuscript was written by A.S. All authors contributed to the final manuscript.

## Declaration of interests

The authors declare no competing interests.

## References

[1] Amalamol D., Ashwin N.M.R., Lakshana K.V., Nirmal Bharathi M., Ramesh Sundar A., Sukumaran R.K., Malathi P., Viswanathan R. (2022). A highly efficient stratagem for protoplast isolation and genetic transformation in filamentous fungus Colletotrichum falcatum. Folia Microbiol..

[2] Turgeon B.G., Condon B., Liu J., Zhang N. (2010). Protoplast Transformation of Filamentous Fungi. Methods Mol. Biol..

[3] Zou G., Zhou Z. (2021). CRISPR/Cas9-Mediated Genome Editing of Trichoderma reesei. Methods Mol. Biol..

[4] Lyu Y., Wu P., Zhou J., Yu Y., Lu H. (2021). Protoplast transformation of *Kluyveromyces marxianus*. Biotechnol. J..

[5] Lim F.-H., Rasid O.A., Idris A.S., As’wad A.W.M., Vadamalai G., Parveez G.K.A., Wong M.-Y. (2021). Enhanced polyethylene glycol (PEG)–mediated protoplast transformation system for the phytopathogenic fungus, Ganoderma boninense. Folia Microbiol..

[6] Allen M.F., Shulman H., Rundel P.W., Harmon T.C., Aronson E.L. (2023). Leaf-cutter ants – mycorrhizal fungi: observations and research questions from an unexpected mutualism. Front. Fungal Biol..

[7] Veličković M., Wu R., Gao Y., Thairu M.W., Veličković D., Munoz N., Clendinen C.S., Bilbao A., Chu R.K., Lalli P.M. (2024). Mapping microhabitats of lignocellulose decomposition by a microbial consortium. Nat. Chem. Biol..

[8] Folgarait P.J., Goffré D. (2023). Control of pest ants by pathogenic fungi: state of the art. Front. Fungal Biol..

